# Systematic review on the evidences of an association between tinnitus and depression

**DOI:** 10.5935/1808-8694.20130018

**Published:** 2015-10-14

**Authors:** Luciana Geocze, Samantha Mucci, Denise Caluta Abranches, Mario Alfredo de Marco, Norma de Oliveira Penido

**Affiliations:** MSc; PhD student. Psychologist - Medical School of the Federal University of São Paulo -UNIFESP-EPM; PhD. Post-Doctoral program. DDS - UNIFESP-EPM; PhD. Associate Professor of Psychiatry - UNIFESP-EPM; Post-Doctoral Degree - Adjunct Professor of Otorhinolaryngology - UNIFESP-EPM. Department of Otorhinolaryngology and Head and Neck Surgery, Federal University of São Paulo - UNIFESP-EPM

**Keywords:** depression, review, symptoms, tinnitus

## Abstract

Tinnitus has been associated with several psychiatric disorders, however there are still several questions regarding such association.

**Objective:**

To assess the scientific evidence on the associations between symptoms of depression, depression, and tinnitus.

**Method:**

A systematic review was performed using PubMed, Lilacs, and SciELO scientific databases. This review included studies published in Portuguese, Spanish, or English correlating tinnitus with depression; letters to the editor and case reports were excluded.

**Results:**

A total of 64 studies were identified, of which only 20 met the inclusion criteria and only 2 were case-control clinical trials. The majority of the studies (n = 18) found that depression is associated with tinnitus, either as a predisposition - resulting in poor adaptation to tinnitus or as a consequence of severe disease.

**Conclusion:**

An overall assessment of all of the selected studies suggests at least 3 possible associations between depression and tinnitus: depression affecting tinnitus, tinnitus predisposing individuals to depression, and tinnitus appearing as a comorbidity in patients with depression. There is a high prevalence of depressive symptoms in individuals with tinnitus, but the mechanisms by which depression and tinnitus mutually interact, are not fully understood.

## INTRODUCTION

Tinnitus is a symptom defined as an auditory perception in the absence of an external source of sound^1^^,^^2^. According to the World Health Organization (WHO), 278 million people have tinnitus - approximately 15% of the world population. This prevalence increases to 33% among individuals with more than 60 years of age^2^, ^3^, ^4^. In Brazil, it is estimated that we have 28 million individuals with tinnitus, making it a public health problem^5^.

Jastreboff^3^ and Sanchez et al.^6^ reported that 20% of the patients with tinnitus consider it a significant nuisance with a negative impact in their lives, which may cause depression and, in extreme cases, even suicide. Hallam et al.^7^ stress that the lack of habituation in some patients with tinnitus may be associated with personality traits or symptoms of depression.

The diagnosis of depression implies in a clear change in mood and depression symptoms. These alterations must last for at least two weeks and have a considerable impact on the work and family affairs of the individual^8^.

Depression may be associated with an increase in the difficulty to adapt to the compromises brought about by a chronic disease. There are indications that depression symptoms, as well as depression itself, are associated with such increase^9^^,^^10^. It is highly prevalent, with estimates that it affects 3% to 5% of the general population. In clinical settings, the incidence is even higher, since depression can be found in 5% to 10% of outpatients and in 9% to 16% of hospitalized patients^11^.

We know that depression is frequent in almost all chronic diseases, and when present, it leads to a worsening in quality of life, worse disease progression and worse compliance to treatment^12^.

Depression symptoms are: sadness, melancholy, frequent sobbing, apathy, a feeling of boredom, increased irritability, a feeling of hopelessness, lack of enthusiasm, insomnia or hypersomnia, loss or increase in appetite, libido reduction, anhedonia, ideas of death, pessimism, ideas of regret and guilt, suicidal acts or ideation, attention deficit, memory deficit, difficulty making decisions, a feeling of incapacity, slow thinking, psychomotor slowing down, negativism, ideas of ruination, hypochondriac delirium, auditory or visual delusions^8^.

The literature has studies concerning the presence of psychopathological factors associated with tinnitus. Tinnitus has been associated with numerous psychological problems, psychosomatic and psychiatric disorders, as well, especially, mood disorders. Depression symptoms are common in individuals with tinnitus, and may worsen their suffering. The mechanisms through which depression interacts with tinnitus are not thoroughly understood, but is a strong association with depression in patients with tinnitus^13^.

The nuisance associated with tinnitus is subjective and variable in relation to its intensity and frequency, and it might compromise the patient's life in a global way, causing personal, professional, social and family impairments.

The goal of this study is to establish the scientific evidence of the association between depression symptoms, depression and tinnitus.

## METHOD

We carried out a systematic review of the papers published on tinnitus and depression indexed in the following databases: PubMed, SciELO and LILACS.

Using the keywords taken from the Medical Subject Headings (MeSH), we setup the following advanced search strategy in PubMed: “Tinnitus”[Mesh] AND “Depressive Disorder”[Mesh] AND (“humans”[MeSH Terms] AND (English[lang] OR Spanish[lang] OR Portuguese[lang]) AND “adult”[MeSH Terms]).

In the LILACS and SciELO databases, we utilized the keywords indexed in the Health and Sciences Keywords and we setup the following strategy: *Tinnitus AND Depre*$. We used the basic research form with the term *AND* in order to select the words (*depression and tinnitus*) and the trunking sign $ in order to search for words with the same keyword root “depression”.

The inclusion criteria were studied in patients older than 18 years, published in English, Portuguese or Spanish, associating tinnitus and depression. We took off letters to the editor and case studies.

Data extraction from the selected papers was carried out by two reviewers, plotted according to the following information: authors, publishing year, sample size, study design, instruments utilized, main outcomes of the association between tinnitus and depression.

## RESULTS

Of the 64 studies found, 53 were found in PubMed, 11 in Lilacs and nine in SciELO; of the Lilacs/SciELO papers, nine were common to the two databases, and two were found in the Lilacs only, making up a total of 64 papers. The papers found are from the last 29 years (1982-2011).

Access to papers was possible thanks to the online distribution through the CAPES website, by the researchers going to the BIREME for access to the printed journals and by response to the authors' request by e-mail or regular mail.

Of the papers found, 20^14^, ^15^, ^16^, ^17^, ^18^, ^19^, ^20^, ^21^, ^22^, ^23^, ^24^, ^25^, ^26^, ^27^, ^28^, ^29^, ^30^, ^31^, ^32^, ^33^ met the criterion to participate in the systematic review, and were included in the study; and 44 were taken off for being case reports, letters to the editor or for not correlating depression symptoms or depression with tinnitus.

Of the 20 studies matching the inclusion criteria, 12 were cross-sectional^14^, ^15^, ^16^, ^17^, ^18^, ^19^, ^20^, ^21^, ^22^, ^23^, ^24^^,^^29^, three were prospective and longitudinal^25^, ^26^, ^27^, three were case-controlled^28^^,^^30^^,^^31^ and two were clinical cases^32^^,^^33^.

A positive correlation between tinnitus and depression was found in 18 studies^16^, ^17^, ^18^, ^19^, ^20^, ^21^, ^22^, ^23^, ^24^, ^25^, ^26^, ^27^, ^28^, ^29^, ^30^, ^31^, ^32^, ^33^, that is, these studies proved that depression is somehow associated, predisposing the patient to a maladaptation to tinnitus or as a consequence of the tinnitus severity. However, two studies^14^^,^^15^ did not find any significant association between depression and tinnitus, these two were cross-sectional studies.

The most used scale to detect depression was the BDI (eight studies)^14^^,^^15^^,^^18^^,^^19^^,^^21^^,^^23^^,^^24^^,^^31^ and the one most used to detect depression symptoms was the HADS (seven studies) ^22^^,^^25^^,^^27^^,^^29^^,^^30^^,^^32^^,^^33^, almost always associated with a severity scale of tinnitus symptoms, and the THI (five studies)^14^, ^15^, ^16^^,^^23^^,^^27^ the most used instrument. The samples varied much in size, from 27 to 1275 patients, with a mean value of 167.3 (Chart 1).

**Chart 1:** Description of the papers matching inclusion criteria.

Authors and year	Study type	Sample	Instruments	Main results of Tinnitus × Depression
Ooms et al. 2011^14^	Cross-sectional	136	BDI^a^, THI^b^	None or minimum depression symptoms. It was only the BDI somatic subscale which was associated with tinnitus severity.
Figueiredo et al. 2010^15^	Cross-sectional	48	THI, BDI	The incidence of depression in the sample was low (8.3%), weak correlation between tinnitus and depression.
Mathias et al. 2011^16^	Cross-sectional	50	MINI v.5.0^c^ e THI	82% of the patients had some psychiatric disorder, 40% had major depression.
Simpson et al. 1988^17^	Cross-sectional	41	SCID^d^	The group with tinnitus had a predominance of mood disorders (46%), and 1/3 were depression disorders.
Kirsch et al. 1989^18^	Cross-sectional	77	BDI, PSC^e^, LES^f^	The group with less adaptation to tinnitus nuisance was the one with the most depression symptoms. When compared to the group without tinnitus, the BDI score was twice higher in the patients with tinnitus.
Budd et al. 1995^19^	Cross-sectional	109	BDI, STAI^g^, TS^h^, LCB^i^	The correlations between BDI and tinnitus severity remained significant, BDI was significantly correlated with tinnitus severity.
Hiller et al. 1997^20^	Cross-sectional	1275	SSD^j^, SDS^k^	11% stated they had tinnitus, and from these, 45 (3.5% of the total sample) had depression.
Folmer et al. 2001^24^	Cross-sectional	160	TSI^l^, aBDI^m^	Tinnitus severity was associated with insomnia, stress and depression. 31% reported having depression, 35% had prior history of depression, 21% had scores for major depression.
Andersson et al. 2003^22^	Cross-sectional	157	HADS^n^, TRQ°, ASI^p^	17% prevalence of depression in the Internet sample and 15% in the clinical sample.
Langguth et al. 2007^23^	Cross-sectional	100	THI, TQ^q^, BDI	Significant correlation between tinnitus severity and depression. 20.8% severe or moderate symptoms of depression, and 34.7% mild symptoms.
Folmer et al. 2008^21^	Cross-sectional	200	TSI, aBDI, SAI^r^	Positive correlation between OCD, depression and tinnitus. 49% symptoms of depression, 27% moderate or severe depression.
Holdefer, et al. 2010^25^	Prospective cohort	27	THI e HADS	THI results before and after group therapy were: functional 29 and 14; emotional, 24 and 10; and catastrophic, 12 and 5, respectively; HADS scale: stress, 12 and 9; and depression, 10 and 6.
Holgers et al. 2005^26^	Prospective cohort	127	TSQ^s^, SCID	SCID-P showed a high comorbidity of depression disorders in patients with tinnitus.
Westin et al. 2008^27^	Prospective cohort	47	TAQ^t^, THI, HADS	High correlation between depression and quality of life in the follow up, and partial correlation for the tinnitus nuisance.
Sullivan et al. 1988^28^	Case control	40	NIMH DIS^u^, SCL-90^v^	78% of the patients with tinnitus had one or more episodes of major depression, compared to 21% in the control group; 60% of the patients with tinnitus had major depression at the time of the interview, compared to 1 (7%) control individual.
Sullivan et al. 1994^29^	Cross-sectional	92	THQ^w^, HADS	The best predictor of maladaptation was depression.
Zöger et al. 2006^30^	Case control	224	TSQ, SCID, HADS, CPRS-S-A^x^	39% had minor depression and 33%, a major depression.
Hébert et al. 2007^31^	Case control	102	BDI, TRQ	Patients with tinnitus had more depression symptoms, although the mean values were within normal ranges.
Sullivan et al. 1989^32^	Non-randomized clinical trial	19	THQ, HADS	Strong correlation between severe tinnitus and depression symptoms, pointing to a bidirectional association.
Sullivan et al. 1993^33^	Randomized clinical trial	92	HADS, TDM^y^	Antidepressants reduces depression, functional incapacity and tinnitus intensity.

a Beck Depression Inventory;

b Tinnitus Handicap Inventory;

c Mini International Neuropsychiatric Interview (version 5.0);

d Structured Clinical Interview for DSM Disorders;

e Psychosomatic Symptom Checklist;

f Holmes and Rahe Life Events Scale;

g State-Trait Anxiety Inventory;

h Tinnitus Severity;

i Locus of Control of Behaviour;

j Screener for Somatoform Disorders;

k Somatoform Disorders Schedule;

l Tinnitus Severity Índex;

m Abbreviated version of the Beck Depression Inventory;

n Hospital Anxiety and Depression Scale;

• Tinnitus Reaction Questionnaire;

p Anxiety Sensitivity Índex;

q Tinnitus Questionary;

r State Anxiety Inventory;

s Tinnitus Severity Questionnaire;

t Tinnitus Acceptance Questionnaire;

u Mental Health Diagnostic Interview Schedule;

v Hopkins Symptom Checklist;

w Tinnitus Handicap Questionaire;

x Comprehensive Psychopathological Rating Scale;

y Tinnitus Disability Measures.

Two cross-sectional studies were unable to establish a significant correlation between depression and tinni-tus^14^^,^^15^. In a study carried out by Figueiredo et al.^15^, the low incidence of depression (8.3%) in the study sample, showed a weak correlation between tinnitus and depression. One explanation could be the fact that the THI score was below the expected mean value and, in the literature, depression is associated to an increase in the nuisance, high THI scores. Ooms et al.^14^ found none or a minimum presence of depression symptoms. However, there was a significantly positive correlation (*p*< 0.01) between the presence of somatic symptoms in the depression scale and the nuisance of tinnitus by the THI, capable of indicating an association of somatic symptoms and the severity of tinnitus.

Three prospective and longitudinal studies showed a high comorbidity of depression disorders in tinnitus patients^25^, ^26^, ^27^. Holdefer et al. showed that the severity of tinnitus can significantly predict depression in the follow-up^25^. In the study carried out by Holgers et al.^26^, there was a high prevalence of depression disorders in patients with severe tinnitus, and the study carried out by Westin et al. showed that the symptoms of depression, was the only measure with significant results in the follow-up^27^.

Three case-control studies^28^^,^^30^^,^^31^ also showed a positive correlation between depression and tinnitus. Hébert et al.^31^ compared the prevalence and severity of 27 physical symptoms in 51 patients with major depression and 51 paired controls, and found that 49% of the patients with depression also complained of tinnitus, compared to 11.8% of the controls. In the study carried out by Zöger et al.^30^, there was a correlation between tinnitus severity and depression, 39% of the patients had depression and 33% had major depression, and they concluded that depression is the most important factor associated to suffering in patients with tinnitus and was responsible for approximately 20% of the variance observed in the association. Sullivan et al.^28^ reported that patients with tinnitus had a significant prevalence of major depression during life (78% *vs*. 21%) and significantly higher prevalence of depression at the time of the study (60% *vs*. 7%) when compared to controls.

## DISCUSSION

The most relevant studies found were the two clinical trials. Sullivan et al.^32^ carried out a non-randomized, bind, placebo-washout clinical trial using nortryptiline, with 19 patients with severe tinnitus, diagnosed with major depression and concluded that treatment with antidepressants reduces tinnitus severity, alleviating its nuisance, since it reduced the intensity of depression symptoms. And Sullivan et al.^33^ did a randomized, double blind, placebo-controlled clinical trial using nortriptyline with 92 chronic tinnitus patients, 38 patients diagnosed with major depression and 54 patients had depression symptoms and tinnitus nuisance. They reported an improvement in depression symptoms and of the tinnitus nuisance; however, the patients considered the side effects of the medication as harmful to their quality of life. The authors suggest a strong correlation between severe tinnitus and depression symptoms a bidirectional association. Tinnitus can cause an increase in depression symptoms - which can cause a poor adaptation to tinnitus and, consequently, increase the tinnitus severity. They concluded that the treatment with antidepressants for patients with severe chronic tinnitus may be considered useful, but we still need clinical trials involving non-pharmacological treatments for depression, correlating with the quality of life of these patients.

There are at least three possibilities of associating depression and tinnitus: depression as one of the factors which worsens tinnitus^18^^,^^19^^,^^22^^,^^28^^,^^31^, tinnitus as a predisposing factor to depression^14^, ^15^, ^16^, ^17^^,^^20^^,^^24^ and tinnitus installing as a comorbidity in patients with depression^18^^,^^26^, ^27^, ^28^^,^^32^; in such case, depression increase the intensity, the discomfort and the intolerance to tinnitus, increasing depression.

## CONCLUSION

There is a high prevalence of depression symptoms in patients with tinnitus. Studies suggest at least three possible associations between depression and tinnitus: depression as a factor affecting tinnitus, tinnitus as a factor which may predispose individuals to depression, and tinnitus as a comorbidity in patients with depression. However, the mechanisms of interaction between depression and tinnitus are not fully understood. Future studies are necessary to help unveil the cause-and-effect association between depression and tinnitus.
